# Beyond individualism: Is there a place for relational autonomy in clinical practice and research?

**DOI:** 10.1177/1477750917704156

**Published:** 2017-04-13

**Authors:** Edward S Dove, Susan E Kelly, Federica Lucivero, Mavis Machirori, Sandi Dheensa, Barbara Prainsack

**Affiliations:** 1 J. Kenyon Mason Institute for Medicine, Life Sciences and the Law, School of Law, University of Edinburgh, UK; 2 Department of Sociology, Philosophy and Anthropology, College of Social Sciences and International Studies, University of Exeter, UK; 3 Department of Global Health & Social Medicine, Faculty of Social Science & Public Policy, King’s College London, UK; 4 Faculty of Nursing and Midwifery, King’s College London, UK; 5 Clinical Ethics and Law, Faculty of Medicine, University of Southampton, UK

**Keywords:** Autonomy, care, consent, ethics, healthcare, individualism, relational autonomy

## Abstract

The dominant, individualistic understanding of autonomy that features in clinical practice and research is underpinned by the idea that people are, in their ideal form, independent, self-interested and rational gain-maximising decision-makers. In recent decades, this paradigm has been challenged from various disciplinary and intellectual directions. Proponents of ‘relational autonomy’ in particular have argued that people’s identities, needs, interests – and indeed autonomy – are always also shaped by their relations to others. Yet, despite the pronounced and nuanced critique directed at an individualistic understanding of autonomy, this critique has had very little effect on ethical and legal instruments in clinical practice and research so far. In this article, we use four case studies to explore to what extent, if at all, relational autonomy can provide solutions to ethical and practical problems in clinical practice and research. We conclude that certain forms of relational autonomy can have a tangible and positive impact on clinical practice and research. These solutions leave the ultimate decision to the person most affected, but encourage and facilitate the consideration of this person’s care and responsibility for connected others.

## Introduction

Respect for autonomy is one of the most important values guiding clinical practice and research in many parts of the world.^1,a^ At the same time, it is closely connected to a particularly Western, post-Enlightenment idea that an adult person is a bounded individual who is able to live her life freely in accordance with her self-chosen plan, and ideally *independently* from controlling influences.^2–8^ In the field of medicine, the principle of personal autonomy was raised as a pillar in clinical research ethics beginning in the mid-20th century. This was, to a large extent, a response to the Nazi atrocities in the name of medical research. It is reflected, for example, in the Nuremberg Code and especially its first point that the ‘voluntary consent of the human subject is absolutely essential’, with ‘sufficient knowledge and comprehension of the elements of the subject matter involved’ so as ‘to enable him to make an understanding and enlightened decision’.^9^ In the domain of Western biomedicine, the epitome of personal autonomy is a patient expressing a decision that she has come to autonomously and independently. The importance of such an understanding of autonomy – which we call ‘individualistic autonomy’ – within law and clinical ethics is summarised in the words of the respected American judge, Benjamin Cardozo: ‘Every human being of adult years and sound mind has a right to determine what shall be done with his body; and a surgeon who performs an operation without his patient’s consent, commits an assault’.^10^

Many within and outside of the field of medicine see such an individualistic understanding of autonomy as a positive development, ‘serv[ing] as a corrective to the highly paternalistic doctor-patient relationship’^11^ that has dominated the history of Western medicine.^12^ Besides its constitutive role in conceptualising personhood in the West, individualistic autonomy has practical advantages, especially for law. As King and Moulton argue, by establishing a seemingly clear rule for how and by whom decisions should be made (i.e. by the affected patient/participant), individualistic autonomy provides a seemingly simple recipe for the protection of patient autonomy. Such an easy-to-follow recipe eases burdens on healthcare professionals in several ways:

1) protecting autonomy is more easily aligned with existing legal principles and precedents; 2) promoting patient autonomy may relieve the physician of some responsibility and liability; 3) emphasizing patient autonomy coincides with and supports the recent shift towards consumerism in medicine; and 4) promoting autonomy appears less paternalistic than beneficence, but still permits physicians to control the flow of information.^13^

This said, the individualistic conception of autonomy as the self-rule of independent, self-determining and rational (in the sense of strategic rationality) individuals has faced theoretical challenge in recent decades.^14,15^ One strand of critique has been posited by feminist and communitarian scholars, who argue that an individualistic understanding of autonomy is both insufficient to capture the breadth of human interests and agency, and inconsistent with other important values.^3,16–20^ Critics argue that understandings of autonomy should accommodate the fact that people are rarely, if ever, fully independent individuals. Instead, we are relational beings whose identities and interests are shaped by our connections to others. In other words, it is through relations to our human, natural and artefactual environments that we come to develop our sense of identity as well as capacity for exercising self-determination. Some scholars advocating such a relational conception of autonomy further posit that the individualistic conception causes tangible problems in healthcare and research.^21,22^ These problems range from legal and ethical barriers to data use, to increasing suffering for patients at the end of their lives.^23,24^

Interestingly, the deep and nuanced theoretical contributions that challenge such an individualistic conception of autonomy and formulate alternatives have had little impact on actual clinical practice and research. Looking at the English legal context in healthcare, for instance, Gilbar and Miola observe that ‘the emergence of relational autonomy has not yet yielded a meaningful legal response to the impact of the patient’s cultural background on the decision-making process, thus creating difficulties for clinicians and some patients to make decisions about treatment’.^25^ Similarly, McLean notes the law’s ongoing strong focus on the individual’s decision-making capacity and her *individual right* thereof to exercise in decision-making.^26^ Despite her criticisms of the law’s extant approach, however, McLean is equally concerned that emphasising ‘the importance of inter-relatedness can result in an undue focus on the interest of others and this is evident, for example, when the decisions of pregnant women are disregarded in the interests of a future child’. Placed in the ‘wrong’ hands, relational accounts ‘can be manipulated to defeat autonomous choice’,^26^ meaning that, in McLean’s specific example, it unjustly overrules the interests of the pregnant woman.

The fear of infringing individual rights and interests by employing a different understanding of autonomy may be an important reason for the fact that the wide-ranging and nuanced literature on relational autonomy has remained without much impact on practice so far. But are there additional reasons for the gap between theory and practice? Is relational autonomy a notion that compels in the abstract but fails the test of practice? Or can relational autonomy help to address some of the challenges that clinical practice and research are facing, and if so, how? These are the questions that the remainder of this article seeks to address. As a growing number of bioethicists and social scientists invoke a ‘relational turn’ that encourages us to move beyond individualistic towards more relational and/or collective perspectives,^27–31^ the time is ripe to put relational autonomy to the test, exploring what it can do when applied to clinical practice and research. In so doing, we emphasise our support of autonomy as a fundamental ethical principle, albeit one that requires reconfiguration. First, we outline the individualistic conception of autonomy and then contrast this with a summary of the theoretical challenge posited by scholars advocating relational autonomy. Observing that instances of operationalising relational autonomy in clinical practice and research are few, we then present four case studies and discuss whether (and if so, how) relational autonomy can change the way we think about and address practical challenges.

## Individualistic notions of autonomy

The individualistic notion of autonomy as enlightened self-determination, first developed by John Stuart Mill in his 1869 essay *On Liberty*,^32^ was famously expressed by Isaiah Berlin as follows:

I wish my life and decisions to depend on myself, not on external forces of whatever kind. I wish to be the instrument of my own, not of other men’s, acts of will. I wish to be a subject, not an object; to be moved by reasons, by conscious purposes, which are my own, not by causes which affect me, as it were, from outside.^33^

Common understandings of autonomy in bioethics are strongly influenced by this individualistic notion, not least because some of the earliest and most powerful institutions of bioethics were born in the heartland of individualistic autonomy – the United States.^34,35^ Because of this strong emphasis on individual independence in the understanding of personal autonomy within bioethics, negative freedom – namely the freedom from interference by others^33^ – plays a central role in its conceptualisation, instead of the ‘positive’ factors and circumstances that need to be in place for people to lead healthy, dignified and meaningful lives.

Such a ‘negative’ understanding of autonomy is fundamentally a device to protect individuals from intrusion by others,^33^ which in turn reinforces the notion that people are independent decision-makers. In practice, this tends to take the form of minimal or ‘thin’ autonomy, where the mere ability to exercise individual choice is taken to be autonomous choice.

In the context of clinical practice and research, respect for autonomy is connected closely to the value of privacy and notion, or practice, of consent.^36^ As Onora O’Neill observes: ‘For proponents of autonomy rights for patients, the physician’s obligations to the patient of disclosure, seeking consent, confidentiality, and privacy are established primarily (and perhaps exclusively) by the principle of respect for autonomy’.^37^ Voluntary, informed consent acts as the process by which one (legally) autonomous individual allows or refuses another individual to perform an action on or relating to her.

But some authors have challenged the strong link between consent and autonomy, though not always explicitly, through the prism of individualism versus relationality.^38^ Because a person can fail to give her consent to a certain course of treatment and yet still be fully autonomous with respect to her decision to pursue the course of treatment, it can be argued that the ethical foundation of consent is really concern for human well-being, though the case for medical paternalism gains strength in arguing this position.

The law, particularly in the context of judicial decisions, tends to focus on other areas of concern, including policy and the desire to balance the interests of patients or participants with other parties (namely healthcare professionals). As such, it often espouses a generalised, objective standard of the ‘reasonable patient’ – asking what information is necessary for an average ‘reasonable’ patient, rather than a specific patient – to make an enlightened decision concerning the medical procedure at hand.^26,39^ This said, the recent UK Supreme Court decision of *Montgomery v Lanarkshire Health Board* acknowledges that doctors have a duty to take reasonable care to ensure a patient is aware of any ‘material risks’ involved in any recommended treatment.^40^ ‘Material risks’ can refer either: (a) to risks that a reasonable person in the position of the patient would likely attach significance to, or (b) to risks that the doctor should reasonably be aware that the *particular* patient would be likely to attach significance to.^41^ Despite greater judicial recognition of subjective notions of risk, the *Montgomery* decision still grounds the issue of consent vis-a-vis the individual, specific patient and her decision-making ability. As McLean observes:

It is the decision-making aspect of autonomy that dominates in law; (legally defined) decision-making ability predicts the status of competence and thereby the right to act autonomously. The individual is supreme, and once judged competent is entitled to make decisions on the basis of his or her own concerns and interests, subject only to the caveat that they do not harm third parties. This individualistic model of autonomy is largely unconcerned with what the decision is; rather, it is interested in the right to make it.^26^

Moreover, the law’s focus on consent is on assessing the individual’s *status* as *competent* (again in a legal sense), rather than on dialogical and iterative mechanisms that engage the person to assess the background psychological aspects that assure information is complete enough to enable an informed, ‘autonomous’ decision. Consequently, ‘shared decision-making’ that values the dialogue between healthcare professionals and patients or participants – and others – in the consent process is side-lined in the law of many jurisdictions. Instead, a formulaic approach to consent predominates, assuming that patients can be given information and then make an independent decision on the basis of this information. As Spatz and colleagues observe in the American context (but which can be applied to many other jurisdictions as well):

[W]ith the exception of 1 state, Washington, that explicitly recognizes shared decision making as an alternative to the traditional consent process, the law has yet to promote a process that truly supports a reasonable-patient–centered standard through shared decision making.^39^

The legal position reflects the emphasis that an individualistic understanding of autonomy places on who counts as an autonomous person and the procedural conditions of exercising choice, rather than on what constitutes a good decision. Proponents of individualistic autonomy value the *assertion and exercise* of choice to do something, for example, to participate in a research study or to agree to have surgery performed. In the words of Katri Lõhmus:

This form of autonomy pays attention to the procedural conditions of one’s choices, *how* a decision is made rather than *what* is decided. As long as certain necessary conditions on the decision-making process are in place, the choice counts as autonomous, regardless of the value (or lack of value) of the object chosen. As a result, the primary concern and focus for this type of autonomy shifts to the chooser – we have to be deemed competent to make autonomous choices.^42^

Moreover, consent is generally seen – in the ‘default’ setting – as a decision that is and should be expressed by one person, namely the patient or research participant.^24^ To illustrate this, the current version of the Declaration of Helsinki states at Article 25 that: ‘Participation by individuals capable of giving informed consent as subjects in medical research must be voluntary. Although it may be appropriate to consult family members or community leaders, no individual capable of giving informed consent may be enrolled in a research study unless he or she freely agrees’.^43^ This statement acknowledges that a person’s interests are connected to other people and groups and could thus be read as a call for a degree of relational autonomy. But equally it can be seen to underscore individualistic autonomy, suggesting that although others may have interests, it is ultimately only the individual’s decision (expressed through free agreement) that matters. This observation of the ‘default’ setting of personal autonomy is not a trivial point, as it highlights, again, the close relation between the idea of consent and Western individualism.^8^

## Relational autonomy

In recent decades, the individualistic conception of autonomy as atomistic and rational ‘self-rule’ has come under theoretical challenge, particularly by scholars advocating a more relational form of autonomy. Not independence, but *inter*dependence, is at the heart of relational notions of autonomy: social surroundings and relationships enable us to flourish and develop a robust capacity for self-determination and identity formation.

Feminist and communitarian scholars in particular have taken issue with *individualistic*, that is, atomistic autonomy – rather than autonomy itself – for ignoring values such as mutual responsibility, cooperation and care towards others – values seen as playing a crucial role in important areas of decision-making such as healthcare and research. Individualistic autonomy, in these scholars’ view, offers an impoverished or incomplete view of the human condition. Instead, they have encouraged us to reconceptualise what autonomy means, and therefore what it demands from us and others. Relational autonomy can be viewed as a conception of autonomy that places the individual in a socially embedded *network* of others. Relationships (with family, community and society), responsibility, care and interdependence are key attributes of relational autonomy: people develop their sense of self and form capacities and life plans through the relationships they forge on a daily and long-term basis. Relational autonomy asserts, therefore, that social surroundings and relationships are crucial for developing autonomy, and encourages us to act in ways guided by an ethic of trust and care.^44,45^

This said, it remains unclear what exactly constitutes a ‘relational autonomy response’ to individualistic autonomy in practice. Two important reasons for this may be lack of consensus about the analytic and normative value of relationality, and difficulties regarding the question of how to achieve the consensus. Most people would consider social relations to be important for human flourishing; not even the fiercest supporters of the rational choice paradigm and individualistic autonomy would deny that. Where views diverge, however, is the question of whether we should treat the statement that ‘relations are important to human beings’ merely as the description of a social fact, or whether it should have deeper analytical and also normative implications.^b^ Similarly, just as there are different views on the analytic implications of the importance of relations to others for human beings, positions vary on whether relationality should have normative implications on practice. The ethic of care approach, for example, embodies the position that relationality does have normative implications on how we treat people.^48–52^

The picture is complicated further by the multitude of relational accounts of autonomy. They range from understanding relational autonomy foremost as a reminder for practitioners to critically reflect on the limits of their and other people’s autonomy,^53^ to giving more decisional space to family members and a patient’s or research participant’s significant others in medical decision-making,^54,55^ to approaches that treat relational autonomy as a justification to challenge decisions that people make that seem, for one reason or another, problematic.^29,56^

This said, it is possible to distinguish between two broad strands of thought on relational autonomy.^19^ The so-called ‘causal view’ posits that an individual faces external constraints that cause alterations to her free choice and scope of possible actions. To exercise her autonomy, the individual must remain situated in a web of relations; absent relations, she lacks autonomy. However, in such a causal understanding, autonomy itself need not be determined by social conditions. It would be possible, within this framework, to understand autonomy in an individualistic manner: while social conditions in the background enable autonomy in a more general sense, determining whether someone acts autonomously in a specific situation depends on their internal psychological state or capacities. The ‘constitutive view’, in contrast, does not treat social conditions merely as a background condition for autonomy, but posits that people are directly constituted by their relations and concerns for others.^45^ Here, autonomy is imbricated with interpersonal relations and social conditions. What both of these accounts share is that they posit that conceptions of autonomy *must* pay heed at some level to external social conditions.

But, as mentioned in the Introduction, practical clarity fails to emerge from this theoretical discussion. In the process of making the conceptual work on relational autonomy relevant for practice, the following questions are of key importance: What are the boundaries of a person? Should or must there be any external conditions set around these boundaries? What is ‘external’ to a person if all her relations are somehow part of her? Should we consider only the patients’ family, friends and others as the ‘relations’ relevant to relational autonomy, or does her healthcare professional (e.g. doctor, nurse) – or the researcher – count as well? If so, what would this look like in practice? And what about close relationships that seem abusive or exploitative? Moreover, can relational autonomy be operationalised in law, which at least in the West has been shaped by methodological individualism – one body, one mind, one person? More simply, what *work* can relational autonomy do for us as participants, patients, clinicians, researchers, policymakers and as citizens?

There have been surprisingly few attempts to translate the rich and nuanced theoretical critique of the individualistic notion of individual autonomy into new approaches and tools for decision-making in clinical practice and research. In the following section, we present four case studies as thought experiments, querying whether and how re-thinking the individual by considering the ‘social’ in decision-making processes can allow us to move beyond manifestations of individual autonomy in the healthcare and research context. Given the extant lack of practical clarity, our key question is: in the context of specific healthcare practices, how does a relational autonomy approach help to reframe the existing impasse, and what are the new dilemmas that it raises?

## Case studies: Can relational autonomy help?

### Case 1: Re-contacting patients with new genomic and health findings

The availability of technologies to analyse genetic material quickly and relatively inexpensively raises important issues related to the communication of the potential health significance of new findings, such as new information about the natural history of a condition; improved diagnostics; new information about previously uncertain test results; or reclassification of a variant on the basis of which decisions have been made. Specifically, questions arise about whether healthcare professionals, such as clinical genetics specialists, should re-contact former patients when new findings emerge. No professional consensus has been reached on whether such a responsibility to former patients exists, and how it might enhance or interfere with patient autonomy. Interestingly, in the ethics of clinical genetics literature, the ‘right not to know’ has been developed as an offshoot of (individualistic) autonomy.^57,58^ An individualistic understanding of autonomy sets up a dilemma here in that it suggests a particularly individual-centred understanding of rights and responsibilities that may lead to difficulties for a healthcare practitioner whose view of her own responsibility may conflict with the views of her patient, including her ‘right not to know’. In this case, patients may or may not want to be re-contacted, as individual and family sensitivities may be involved, and respecting their ‘right not to know’ may put the conscientious healthcare professional in an uncomfortable position when he or she feels that certain information should be shared, and remains uncertain what her responsibilities involve.

Because of these difficulties, currently there is no professional consensus in clinical genetics about whether, and how, former patients should be re-contacted when new genetic information relevant to them or their family members comes to light. The only guideline currently available is a statement originally published in 1999 by the American College of Medical Genetics.^59^ This document highlights the logistical difficulties of re-contacting former patients, and identifies the primary care provider – the specialist tasked to provide continuing care, such as a family physician or General Practitioner (GP) – as the principal responsible healthcare provider to alert patients to the potential need for re-contact. Genetic service providers would be responsible for clinical updates to patients in the cases in which they are offering continuing care. The statement also suggests that patients should be appropriately advised to update their primary care provider or the genetic service provider if relevant changes in their lives occur, such as pregnancy.^59^ The 2007 revision of the statement recognises that with the uptake of next generation sequencing, testing laboratories may now be in a position to know about changes in interpretation of variants whose significance had been previously unknown, or about reclassifications of previously classified variants – and should make an effort to contact relevant healthcare providers if new information changes the previous clinical interpretation of a sequence variant.^60^

In addition to not providing an answer to whether, and if so how, re-contacting should be understood as appropriate clinical practice, individualistic notions of autonomy and privacy have not led to practical solutions to these questions.^61^ As Dheensa and colleagues observe, current understandings of information-sharing in genetic medicine are ‘based on an inaccurate conceptualisation of patients as separate from others, free from social or familial constraints’.^30^ Can a relational autonomy approach help here?

The *Mainstreaming Genetics: Re-contacting Patients in a Dynamic Healthcare Environment* project^c^ in the UK has found that patients hold expectations that they will be re-contacted, while healthcare providers express concern about the availability of resources to do so. Further, the project found that most clinical genetics services currently do re-contact patients, but not on a systematic basis.^62^ The project is considering proposing a ‘partnership’ model for responsibility for re-contacting^63^ that respects both the ‘right not to know’ of the former patient (if this is what the patient wants), and the relational autonomy of the patient, which includes relationships with the healthcare provider and health system as well as with family members.

In the partnership model, the clinician consults with her patients at the initiation of the clinical relationship about sharing responsibility to keep up-to-date about new findings and relevant information and communicating that information to biologically relevant others. The point here is not just to record preferences, but to engage in a dialogue about expectations and understandings. In this model, there is reciprocity of rights and responsibilities: the clinician has the responsibility to hold and interpret genetic data and the patient is responsible for ‘triggering’ the request for review of information by maintaining periodic contact with the clinic and informing them of potentially relevant life changes. This means that patients retain control over the possibility of receiving information concerning updates about their condition or genetics, respecting their personal autonomy (which can be understood to include the ‘right not to know’). At the same time, this solution could be seen as employing relational autonomy where patients are seen as embedded within relations that include responsibility and accountability to both themselves and others (including, but not limited to, biological relatives), and where such relations also involve the healthcare professional with whom the parameters of the partnership would be negotiated. The responsibility to communicate information to connected others would be negotiated between the patient and clinician, who share responsibility to consult with connected others in the process of communicating updates, meaning that no ‘one size fits all’ recommendation or guideline can be made. There is reason to argue here that championing a shared decision-making model, as in this partnership approach, follows from a relational approach to autonomy, or at least respects the patient as being autonomous in a relational sense.^64^

This case highlights, and indeed is complicated by, the problem of potentially competing preferences of third parties. The healthcare provider may perceive a duty to warn a third party of a genetic finding relevant to their health, against the wishes of the patient with whom they have negotiated a partnership. What does relational autonomy have to say about a duty to warn third parties?

Relational autonomy recognises that the patient is not only embedded within relations with her healthcare provider and with her potentially interested relatives, but also that her interests and needs and indeed her autonomy are partly shaped by these relations. It also requires that relationality is acknowledged in the partnership negotiations that will take place with the healthcare provider, to both enhance the wellbeing of the patient herself, and to include potential duties to third parties. Relational autonomy does not require the patient to suffer harm in order to respect the preferences of a third party, but rather to understand those preferences and take them into account (as Beauchamp and Childress^1^ discuss in the ‘obligation to rescue’).

### Case 2: Sharing genetic information to benefit patients’ relatives

In clinical genetics, questions about relational autonomy are particularly pertinent because genetic information is, by its very nature, shared, and emblematic of people’s relations to others. Nevertheless, clinicians who order genetic tests frequently encounter patients who have not discussed the possibility of a higher familial risk with family members.^65^ Consider, for example, the following:

A patient, John Smith, has early-onset heart disease. Investigations in a cardiogenetics clinic show that he has Familial Hypercholesterolaemia (FH) and a pathogenic *LDLR* gene mutation, i.e. a change in this gene that has caused his condition. Two years later, Jeff Smith is referred to cardiogenetics because he knows about a family history of heart attacks. He wants to know his risk and that of his children. After providing his family history, the clinician realises that Jeff is biologically related to John, and that the heart attacks in both families might be attributed to FH.

In some cases, patients explicitly refuse to inform family members or to allow clinicians to do so. A more common situation, and one that this case focuses on, is where the patient’s consent (specifically, consent for clinicians sharing information with family members) is not documented or is ambiguous. It may be that the patient was seen some (in this case, two) years ago, at which point they agreed to tell their family, but evidently, did not.^66–68^ The patient, John, might now be unreachable or unwilling to return to clinic and waiting for successful contact could delay care for Jeff. The default position in general medicine in the UK is to maintain individual confidentiality – i.e. refrain from sharing any information unless it is justified in the public interest (e.g. preventing serious and imminent harm to others). However, it is unclear whether FH is sufficiently serious to meet this criterion, especially since carrying the *LDLR* gene does not always result in having the disease, and the harm resulting from the disease is not always immediate, even if risk-reducing interventions might be immediately available.^69^ The clinician in the example thus faces a conflict of normative duties. On one hand, she thinks she ought to respect John’s confidentiality by not sharing with Jeff what she knows. She considers this as protecting John’s autonomy because he has not given specific consent to share the information. On the other hand, by telling Jeff about the risk and offering him a test, she has the chance to prevent harm to Jeff, as he could benefit from an intervention in case of a positive test result.

An alternative approach to the default position is for clinicians to employ a relational understanding of autonomy and a familial approach to confidentiality.^70,71^ Here, *genetic* information (e.g. that there is an *LDLR* mutation in the Smith family) is conceptualised as ‘belonging’ to both the individual patient and the family. It is thus considered as confidential at the familial level. Individual-level information, such as a specific patient’s genetic or clinical diagnosis or disease status (e.g. that John has FH), by contrast, are regarded as confidential to the individual. This two-tiered approach to confidentiality is underpinned by an understanding of autonomy as relational^71^ also in the sense that interests and needs of a patient’s family have bearing on how some information obtained from an individual patient is handled.

In the context of genetic medicine, Roy Gilbar^66^ has made a similar argument. He suggests that, if taking seriously this relational approach, clinicians will talk to patients about their family relationships and try to achieve a good understanding of what these relationships are like – ideally at the time the patient consents for a genetic test. They could clarify then that as a service that offers genetic testing, they would usually share familial information if it could benefit family members.^72–74^ If the clinician then ends up seeing a family member of a previously seen patient, they could share genetic information pertaining to the family without specific consent from the original patient. What’s more, they could do so without disclosing that patient’s particular genetic or clinical diagnosis or disease status. By treating this individual-level information as confidential to the individual – i.e. not as part of the information belonging to the family – they would be respecting personal autonomy.^75,76^

The ethical bases of the familial approach are the principle of beneficence (here: benefit to the family), fairness and reciprocity. That is, clinicians will have asked a patient – John – for information about his relatives’ health to determine his eligibility for a genetic test. Using the information produced by his test (e.g. that there is a heritable risk) would be a way to enact reciprocity towards those relatives who may not have had the opportunity for a test themselves. Giving John veto power over the use of this information would be unfair. The approach emphasises relational values, such as mutual responsibility (as a manifestation of reciprocity), and interdependence, in that a patient’s decision will affect relatives.

The familial approach to confidentiality has received support from patients^30^ who argue that genetic information is not ‘theirs’, that people have a responsibility to their family members, and any concerns about confidentiality are outweighed by the chance to protect others from harm. By contrast, clinicians have been shown to be wary of taking the familial approach, despite encouragement from UK clinical genetics guidelines.^77^ A major reason for their hesitation was a perception that sharing any information would count as a breach of confidentiality and have legal implications. Another concern was that sharing genetic information might lead to a relative inferring the patient’s identity. It was also feared that if a patient had delayed disclosure due to having a poor relationship with a relative, this inference could have an unknown, but potentially damaging, impact on already fragile relationships.^78,79^ Indeed, in some cases, personal diagnoses might be inferred from other circumstances, e.g. from the fact that a specific person was referred to genetic testing in the first place. If the risk of inference is high, a clinician might decide not to share the genetic information. The key difference between this decision as reached via the familial approach, versus the individual approach to confidentiality, is that nondisclosure is a considered decision, rather than a reflex based on an assumption that sharing is harmful or undermines autonomy. A more nuanced and relational approach might also weigh into the balance that long-held ‘secrets’ can reduce family cohesion and wellbeing.^67,80–83^

In sum, the familial approach to confidentiality draws upon a relational approach to autonomy in that it encourages clinicians to see their patients as embedded in a network of others, to critically reflect, together with their patients, on the needs and interests of these others, and to prominently consider values such as reciprocity and interdependence. The familial approach to confidentiality specifically and relational approaches to autonomy more broadly both respond to a question posed earlier in this article: does the fear of infringing individual rights explain why relational autonomy has not had much impact on clinical practice? The answer, at least in the clinical genetics context, appears to be yes. Careful thinking is now required about how to help clinicians engage with familial and relational approaches in a way that does not supersede, but instead, complements the needs and interests of individual patients.

### Case 3: Placental sampling

Medical research seeks to understand the causes of many types of obstetric diseases, including pre-eclampsia, high blood pressure, diabetes, as well as a range of other conditions that could lead to complications of pregnancy or affect the growth and development of the unborn baby.^84–86^ To better understand why and when complications occur, and how best to respond to them, researchers may collect biological samples from women who fit a research study’s eligibility criteria. Good practice dictates that researchers seek the informed consent from each pregnant woman prior to collecting biological samples and relevant data.^87–89^ This consent also extends to collection of placenta and cord samples.

A question arises as to what happens if, during the consent process, a family or couple disagree over whether to ‘donate’ these samples for research. For the technical consent requirements, this is irrelevant, as only the pregnant woman’s view counts. But for the possibility of obtaining meaningful consent in a clinical research setting, it matters a lot. For example, a couple may be approached about a particular obstetric-related research study, where biological samples ideally are to be collected from the pregnant woman and the postpartum placenta and cord blood. In the absence of other ways of assessing and documenting agreement to participate in research, the consent form is generally accepted as an object indicating understanding and willingness to participate in studies. In some studies, one consent form is required to indicate maternal consent into the study and to donate her own biological samples, while a second form is used to indicate consent to ‘donate’ foetal samples. Based on the clinical experience of one of the authors (MM), most pregnant women appear happy enough to consent to participate in the various studies, yet some request time to discuss the issue with their partner before deciding whether to consent to donation of foetal samples to the study. How is a researcher to react when faced with the latter situation?

During one of the regular recruitment discussions, for example, a woman who had indicated interest in taking part, and who signed her consent form, turned to the researcher and said:

So, you want me to sign the baby into the study? Can he [indicating her partner] sign it? And if I can withdraw from the research anytime, does it mean I have to tell my daughter when she is older that she took part in research so she can withdraw if she wants?

A second illustration comes from another discussion with a different woman admitted on the antenatal ward of the hospital. Having read the information leaflet, she then asked: ‘I have no problems taking part, but is it ok if I wait and ask my husband if he is happy for the baby bit [placenta and cord blood] before I sign it? It’s his baby too’.

For the clinical researcher, it can be very difficult in the face of the disagreeing couple or family to respect only the pregnant woman’s wishes. Often a researcher feels a sense of obligation to incorporate the viewpoint of the partner, but it remains unknown as to how exactly this ‘incorporation’ should be instantiated, and there is currently no legal obligation for a researcher to respect the partner’s opinion. In the face of possible family disharmony, how does one balance research and possible dissent? If DNA extraction is part of the research, collecting maternal or foetal DNA could provide more information about a person’s biological connections; in these instances, just how individualistically autonomous should individuals be in making these decisions which draw in connected others? Researchers are presented a moral dilemma with no clear answer provided by regulation or even best practice exemplar. The main problem, here, seems to be that consent is treated as a decision made by only one person – the pregnant woman – when it is very clear in practice that some of these women do not normally decide, or want to decide, by themselves. While it may seem tempting to remedy this situation by requiring the consent of the woman’s partner, this would open up a whole new range of problems: if the consent of both parents-to-be were required, pregnant women might no longer opt for testing against their partner’s will. Moreover, what should be done in cases where a pregnant woman does not have a partner, or does not want to disclose their identity?

A perspective informed by relational autonomy – namely one that poses emphasis on the web of relations that a person is part of – could provide a solution to this dilemma. It would suggest to retain the requirement of formal consent only from the pregnant woman, but to encourage her to discuss this with her partner and other important people in her life if she wishes. Moreover, room for joint decision-making would be built into the consent process. As highlighted in the previous case study, the circumstances in which joint decision-making could be pursued are not easy to determine since the women’s wishes to perhaps *not* involve her partner or significant family members must also be respected.

In a minimal form, the latter could be done by asking the clinical researcher who seeks the consent to discuss with the woman whom, if anyone, she would like to consult when making this decision, and making adequate time for this. A more expansive version of relational autonomy could entail an explicit acknowledgement of the important role that clinicians and researchers have in this process. It would give the clinician the mandate to proactively encourage that women discuss the decision with significant others. One practical step towards this goal would be to provide training to clinicians and researchers about the contingencies, relationality and contextuality of consent, so that they can discuss the relevance of a situation or issue to a patient’s or research participant’s significant others in a confident and nuanced manner, and so that they consider this aspect as equally important as the collection of data and samples. They would also need to be given adequate time and flexibility to have discussions, possibly over a longer period of time, with the patient or participant.

### Case 4: Patient access to medical records

In September 2015, during the UK’s National Health Service (NHS) annual conference, the Secretary of State for Health, Jeremy Hunt, announced that all patients within NHS England will be given access to their full medical records from 2018.^90,91^ According to Hunt, not only will patients be able to read the notes written by their doctors, as well as prescriptions, hospital referrals and medical tests, they will also be able to contribute to their records by uploading information from their mobile phones. This, Hunt argued, will improve healthcare delivery in two ways: it will make patients be more in control of their health and wellbeing, and it will reduce mistakes in their medical records as patients will be able to rectify them. The NHS Patient Online programme is expected to ‘empower patients to take greater control of their own health and wellbeing by increasing online access to services’.^d^ As stated by Hunt, ‘powerful patients need to know about the quality of healthcare being provided, but they also need to be able to harness the many innovations now becoming possible’.^92^ The announcement was met with a number of concerns, mostly regarding the risks of having such sensitive information available outside the clinical context. Some also mentioned the risk that people in abusive relationships could be coerced or manipulated into revealing the content of private conversations with their healthcare professionals to their abusers.^93^

An important difference between the optimistic scenario described by Hunt and the more sceptical one described by his opponents lies in the assumptions they make. Mr Hunt’s vision assumes that patients can and should be in control of their own health through access to their records. These supposedly empowered patients are independent, autonomous and rational subjects who should know what is best for them; access to detailed medical information empowers them.^e^ As Chiapperino and Tengland remark,^96^ NHS England’s rhetoric of empowerment mobilises a set of values including individual responsibility for disease prevention. While the Royal College of General Practitioners considers patients to be a vulnerable group in need of protection,^93^ in Hunt’s scenario, patients are empowered individuals in control of their conditions. The opportunity to access one’s medical records comes with the ability to act on them and take control. Patient autonomy is both a justifying value and a guiding value in Hunt’s scenario. The conception of autonomy underlying this scenario is clearly an individualistic one. It assumes that people are rational individuals making decisions after processing all available information.

When we turn to *actual* practices of patient access, however, a different picture appears.^f^ An example is Eva’s story. Eva is a young woman affected by several chronic conditions who has been accessing her full medical records through the Patient Access system^g^ for several years. Her work regularly brings her to remote rural areas where she does not have access to the internet. One time while away, Eva realised that she would not have any medication left upon returning home; she called her sister, gave her access details and asked her to log in to the system to repeat a prescription for Eva, so that she could pick up the medication as soon as she returned home. Eva expressed some guilt about having done this:

I probably shouldn’t, but I have used it by proxy through her in the past and it’s been useful. […] Obviously you have to trust someone, but it’s been useful in the past for her to go and check things for me.

Later in a research interview carried out by one of us (FL), Eva explained that her concern about giving her sister the log-in details to access her medical records to repeat her prescriptions came from thinking about e-banking where banks repeatedly warn customers not to share their password with anyone – not even between spouses. ‘And they make it a really big thing […] So you get this impression that you shouldn’t share this information with anyone’, she added. The system seemed to be designed for the single user – the patient – to have complete control and access to it. This system design, however, may not accurately reflect the way things work in practice. Eva has been having access to her mother and father’s medical records as well, as they spend part of the year in Spain with very limited access to the internet. It has happened in the past that Eva’s mother phoned her and asked her to log in with her credentials and order her prescribed medicaments. Their medical care, for both Eva and her family, are personal, but not individual matters. In Eva’s account, her medical care and that of her mother are family matters defined by a mutual ethic of care. Other patients see it differently, but for hardly anybody is medical care a purely individual affair.

The very practices that actual users of direct online access to medical records mention as one of the most beneficial aspects of the system are ironically those that the British Medical Association used to disapprove. In a 2014 guidance regarding ‘Access to health records’,^97^ ‘[a] next of kin has no rights of access to medical records’ (section 4.15). The concern here was that if a *healthcare professional* allowed ‘next of kin’ access to patient medical records, this would lead to a breach of confidentiality. The guidance did not consider the option that patients could consciously and autonomously desire to share access with others. Equally, the computer system in Eva’s story seems unable to recognise the possibility for patients to share access, information, and perhaps also their worries with connected others.

Stories like Eva’s do not neatly fit into the stereotype of atomistic individual agents who are – or at least should be – solipsistically in control of her own health. Her story offers insight into complex webs of mutual support, dependence, love, trust, guilt and concern. The autonomy of these patients can only be understood in its social and intimate relationship with others. Such a relational aspect is absent from Hunt’s portrayal of responsible and empowered individual actors and only partially appears in the Royal College of General Practitioners’ scenario of vulnerable individuals who need to be protected by clinicians from potentially abusive relationships.

This issue has been addressed recently by the Royal College of General Practitioners. In late December 2015, they published a new guidance document allowing proxy access to patient online records.^98^ According to the guidance, patients may now request that ‘someone else, usually a family member, close friend or carer’, has online access to their GP records as their proxy ‘to book appointments, order repeat prescriptions for them or to access their detailed care record on their behalf to assist in their care’.^98^ The guidance specifies that patients should be informed about the dangers and risks of *informal* proxy access (as in the case of Eva’s sister who was using Eva’s credentials to access the system). With formal access, in fact, patients have to select specific functions that their proxies are able to perform, such as booking appointments or repeating prescriptions, for example, without having access to detailed care records. They can also monitor their proxies’ activities and withdraw their access. The guidance also specifies that if GPs suspect that patients are consenting to proxy access against their will, they should ascertain the risk of coercion through a discussion with the patient and may eventually refuse to grant access.

Seemingly moving beyond previous individual-centric guidelines and debates, formal proxy access acknowledges relational aspects in care practices as part of patient autonomy rather than as a limitation thereof. Healthcare professionals are encouraged to discuss the possibility of a family member or friend to access the patient’s data and evaluate case-by-case whether relationality should be prioritised to or balanced with a need to protect vulnerable patients in case of coercion and potentially abusive relationships.

Patients who sign up for electronic access to health records have now the possibility to choose whether they want a trusted friend or family member to have access to their records, and if so, what activities to be performed. It could be argued that this recent change in guidance for online access to GP records takes relational autonomy seriously. A relational approach allows, in fact, one to justify current practices of care from family members and friends and acknowledge that they are carriers and conditions for patients’ autonomy. This system enacts relational autonomy by offering the technical possibility for access, and mitigates the risk of abuse of privacy or breach of confidentiality that would limit the autonomy of the patient.

## Discussion

The case studies discussed in this article suggest that relational autonomy can offer different ways of framing an issue, and in some cases, can offer new solutions to practical problems in healthcare and research. In the first case, an understanding of informed consent emerging from an individualistic understanding of autonomy appears to be more hindrance than help in addressing the question of whether and how to re-contact (former) patients at a later point with new information about their genetics or health condition. Reasons for this include that consent cannot, by definition, be fully informed if patients cannot be asked about future scenarios for being re-contacted. Moreover, treating a person’s consent as a decision of only one person, where medical professionals only provide non-directive guidance, does not begin to accommodate the complex and iterative ways in which such decisions are made in practice.^24^ Here, relational autonomy can lead to the adoption of a shared decision-making model, where everybody’s input, and the joint navigating of high levels of uncertainty, are explicitly acknowledged. Relational autonomy, based on an ethic of care and trust, would suggest to include the healthcare professionals in the decision-making processes. Dheensa and colleagues observe that findings from one person’s DNA may have consequences also for genetically related others (e.g. parents, siblings, children): re-contacting is thus not an individual matter alone.^30^ As such, the sensitivities involved in familial situations may make re-contacting undesirable. The partnership model is designed to put the decision concerning what should trigger a re-contacting event in the hands of the patient, *protecting her autonomy*, but also requiring her to consider the needs and interests of family members when making that decision.

Many of these conclusions also apply to Case 2. As more tests that sequence large parts of the genome are conducted, questions about how to appropriately share information will become more prominent: not only because more and more previous test results will be reinterpreted (leading to potential triggers for re-contacting), but also because testing relatives and exploring their signs and symptoms will be increasingly necessary to make sense of genomic findings that have unclear significance. Using an individualistic understanding of autonomy will be a hindrance not only for re-contacting, but also in thinking about good rules for information sharing with family members when patients are hesitant to do so. Like in our first case, the individualistic approach (which in this case results in keeping all information confidential to the individual unless there is specific documented consent to share) overlooks the complexity of consent. It places too much emphasis on the *documentation* of consent, or lack thereof, seeing it as the final arbiter over what should happen in practice. If a relational approach were endorsed and used more widely, it would encourage clinicians to see the consent process as a chance to discuss and explore patients’ familial relationships – and as we go on to suggest in relation to Case 3, encourage patients to speak to family members where appropriate. When faced with a potential non-disclosure request by patients, a relational approach would move clinicians away from reflexively keeping all information confidential to the individual for fear of infringing that individual’s autonomy. Instead, it would mandate that clinicians encourage patients to consider what their family members may need and want. In practice, it would encourage them to separate information relevant to others from purely personal information and share the former unless there are good reasons not to. This approach could lead to familial benefit and enhanced autonomy of the patients’ web of relations.

The dilemma in the third case is similar to the former two. Clinical researchers are legally obliged to obtain consent only from the woman from whom the placenta to be analysed comes, and not from a partner or the other parent of the child. Researchers may confront the dilemma of deciding whether the prospective participant’s consent is sufficient even if nobody else’s perspective has been considered, or deciding upon the circumstances in which the views of significant others should be sought. Relational autonomy could help with these socio-clinical dilemmas. Asking both parents-to-be for their consent does not seem to be a feasible solution as it would raise more issues than it would solve, not the least of which is the pregnant woman’s right to decide what happens to her body, or the potential for the research to generate tension or conflict between partners and between consent and veto. A relational autonomy-based approach, however, could – similar to the first and second cases – explicitly acknowledge that women often do not make such decisions by themselves, and that partners, family members, friends and even clinicians can be important trusted partners in such decision-making. In this case, relational autonomy would also mandate that decision-making on the side of the woman is not rushed, and adequate time is given for her to consult with everybody whom she wants – and that such engagement is encouraged by healthcare and research professionals.

In the fourth case, the stories told by patients who access their medical records online illustrate how their engagement with their personal medical information is not an individualistic and solipsistic practice. On the contrary, some of the greatest benefits seem to manifest around practices of sharing information and acting for others. The initial rules and guidelines around individual access to medical records sat squarely with the actual practice of use; they assumed that medical records can only be personal to one person and that protection of privacy is an unassailable right of individuals. The recently published guidance for formal proxy access resonates with a relational approach and softens some of this absolutist and atomistic thinking. By inviting GPs to ascertain whether there is a risk of coercion for patients by family members or others who want to access their records, and by enabling patients to monitor the access of their proxies to their records, this system enacts a relational autonomy-based approach. At the same time, as instruments are in place for the patient to retain control over their proxies, patients’ control over their personal medical records does not disappear; rather, the patient is empowered to share control through networks of trust and care.

Together, these cases show that in our quest to enhance the practical value of the concept of relational autonomy in healthcare and research, we must be careful not to remove the patient or participant from the centre of decision-making. At the same time, we should acknowledge that the patient’s decision to consent (or refuse) to treatment or research can be *augmented* by facilitating and encouraging that her relations to, and responsibility for, others are considered in decision-making processes. Our case studies do *not* suggest that we should expand consent requirements to others per se, such as family members or community elders – that is, to add the requirement of seeking consent from further individuals who may also be seen as having a stake in the decision. Such a position would undermine the idea that the person who is centrally affected by a decision should typically have the final say in what happens with and to her, or her body, or even her data. As long as this general principle respects all legal exceptions (see below), we believe that it is a critical underpinning of fundamental respect for persons that should not done away with. Moreover, expanding consent or requiring consent to include others (however so defined) undermines the main objective of relational autonomy, which is to foreground the relational aspect of human identities and interests, and not merely to expand the range of individuals who need to give consent to a procedure.^99^ An approach that merely extends consent requirements to other people does not foreground relations but rather presumptions about who the relevant others of a person are.

The version of relational autonomy that we promote here, on the basis of our case studies, acknowledges the importance of a person’s relations to her (human) others in defining who she is and what her needs are; but it does not abolish the central idea in medicine that the person whose body is, literally, at stake, typically needs to have the final say in what is done to her. This, we hasten to add, is a general rather than universal principle; that the person with and in the body who is most affected by the medical procedure or research project should have the final say does not mean to challenge legal provisions regarding exceptions to this principle, such as in instances of public health or threats to the security of others.^h^ Our claim is that relational autonomy can flourish in clinical practice and research where space – in law, policy, and practice – is provided for people to make decisions also on the basis of considering (or being persuaded but not forced to consider) consultation with others. In so doing, it allows us to preserve the person’s decisional freedom but simultaneously to recognise that autonomous decisions are made by people whose identities and interests are always also shaped by their relations to others.

## Conclusion

In this article, we have endeavoured to illustrate a curious contrast between the rich array of theoretical critique of individualistic notions of autonomy and the paucity of alternative forms of autonomy in practice. It seems that there is a ‘translation gap’ between theoretical critique and the development of new approaches and tools for ethical decision-making in clinical practice and research. Thus, we presented four case studies as a way to address a challenging question: what does relational autonomy actually *change* in how we frame specific ethical problems in clinical practice and research, or how we address them?

The case studies indicate that relational autonomy *does* have a practical role to play in clinical practice and research. Specifically, relational autonomy offers greater analytic and normative value than individualistic autonomy by encouraging wider appreciation of the *socially situated* person, whose decisions are shaped by and consequential for society, and whose interests are rarely purely self-interested. Such an account of autonomy promotes decision-making guided by an ethic of care and moral *responsibility* – whereby the person is respected as an individual but also is encouraged, at levels of legal architecture and clinical practice, to take account of her social situation such that she promotes her own flourishing *as well as* the flourishing of her social and natural environment. Whether this re-conceptualisation of autonomy is taken up in practice largely will depend on how we – as practitioners, participants, patients, policymakers and community members – conceive it and what we want it to do. Relational autonomy changes how we envision self-rule. In the face of entrenched individualistic neoliberalism,^100^ there is a growing social sense among some thinkers and practitioners that the extant paradigm of atomistic and rational self-rule must be supplemented with (but not necessarily replaced by) recognition of the realities of our connected, other-regarding selves,^30,31^ emotional and care-oriented dispositions, and the relations we have with those around us. Importantly, it resists a view in which society consists mostly of individuals exercising their autonomy independent of each other; instead, it embraces collective forms of decision-making and oversight in many contexts. The ongoing challenge has been to instantiate this realisation into practice, and to debate the proper contours of or balance between personal choice and societal concern.

Indeed, what we find upon analysing the case studies is that if relational autonomy is conceived as capacity for self-rule that is (in)formed by relations with, care for, and moral responsibility to one’s informational, natural and/or contextual environments, a space is provided for its instantiation in healthcare and research, albeit one that must be continuously negotiated by the polity to determine where its contours should lie. The case studies suggest that although for many of us, our most important others are family, enacting relational autonomy does not mean that we automatically include family members in decision-making as equals to ourselves. Such an approach would risk that the voices of patients or research participants are merely replaced by those of others. We must be vigilant to potential power imbalances and inequity in any autonomy paradigm, and a mere extension of personal autonomy to the familial would render people vulnerable to the effects of such power imbalances. But because most of us simply do not envisage ourselves as independent decision-making machines with inviolable rights to access or control ‘our’ data, tissue or bodies alone, especially when that data, tissue, and even our body has real and symbolic meaning for others, autonomy is most useful as an ethical norm when we recognise that it does not mean simply being left alone to decide. For it is often in these messy grey zones of in-betweenness and hybridity where the impact of our decisions on others is not only contemplated but valued, that the truest expression of self-rule is manifest. Law and ethics alike must be emboldened to acknowledge the messy grey zones of in-betweenness and hybridity, where participants and patients think and act interdependently, compassionately, emotionally, irrationally and rationally, and often, conflictedly.

## Notes

a The other three clinical ethics principles identified by Beauchamp and Childress are, as many readers know, non-maleficence, beneficence and justice.

b Social science scholars who use participatory research designs, interpretive qualitative methods and other tools that foreground personal meaning and social practices instead of studying opinions and behaviours employ the latter approach.^46,47^ The concepts and methodologies that they use are – explicitly or implicitly – shaped by the commitment that the relational nature of human existence must have an effect on how we study the practices, interests and institutions in our societies. Scholars in the aforementioned tradition of rational choice, in contrast, treat social practices as composites of individual behaviour, and the autonomous individual as the paradigmatic analytic unit of social and political action, as well as the appropriate ‘rights-bearing’ agent in law.

c Information on the project can be found here: http://socialsciences.exeter.ac.uk/sociology/research/projects/details/index.php?id=409.

d This slogan can be found with slightly different wording in NHS England communication material (e.g. flyers, websites, slides). See e.g. https://www.england.nhs.uk/ourwork/pe/patient-online/about-the-prog/po-public/ and http://www.ipswichandeastsuffolkccg.nhs.uk/Portals/1/Content/News%20and%20events/Stakeholder%20newsletter/CCGNewsEngage%20Winter15-16%20no13.pdf.

e The Health and Care Act 2012 and the NHS (England) Constitution (last updated in July 2015) emphasise the importance of patients’ participation in healthcare as a way to empower them. The UK government white papers, *Personalised Health and Care 2020*^94^ and *Five Year Forward View*,^95^ restate the importance of patients’ involvement and emphasise the role of digital systems towards this goal.

f These stories have been extrapolated from 25 in-depth interviews conducted by one of us (FL) with patients in a GP surgery in England that actively promotes patients’ registration to the online system for access to their full medical records. Interviews were conducted between June and September 2015.

g Patient Access is one of the online services for patients to access their medical records and other online systems at their local practices (including arranging appointments, repeat medication and secure messages). This service is provided by EMIS Health, which is the main provider of clinical systems for practices in England. Patients can connect to their practice’s EMIS clinical system through Patient Access. Rival clinical computer systems, like SystemOne developed by TPT, offer their own online systems for patients.

h Various legal cases and statutes address exceptional situations where a person’s autonomy may be curtailed (including reasonable expectations of confidentiality), most often for reasons of public health and safety (e.g. threat of serious harm to others). See e.g. in the United States: *Tarasoff v Regents of the University of California*, 131 Cal Rptr 14 (Cal 1976); *Safer v Pack*, 677 A2d 1188 (NJ Super Ct App Div 1996); in the United Kingdom: Public Health (Control of Disease) Act 1984; National Assistance Act 1948.
